# Do children with and without autism spectrum disorder use different visuospatial processing skills to perform the Rey–Osterrieth complex figure test?

**DOI:** 10.1002/aur.2717

**Published:** 2022-04-05

**Authors:** Ramona Cardillo, Rachele Lievore, Irene C. Mammarella

**Affiliations:** ^1^ Department of Developmental and Social Psychology University of Padova Padova

**Keywords:** autism spectrum disorder, local/global processing, Rey–Osterrieth complex figure, visuospatial organization abilities, visuospatial skills

## Abstract

Visuospatial organization abilities are closely related to other visuospatial processing skills, such as visuomotor coordination, perceptual abilities, mental rotation, and working memory (WM). One task that enables visuospatial organization abilities to be investigated is the Rey–Osterrieth complex figure test (ROCFT). When examining visuospatial functioning, individuals with autism spectrum disorder (ASD) have proved capable of operating both locally and globally, depending on the sub‐domain embraced, with a preference for a locally‐oriented processing of visuospatial information. The present research aimed to establish whether different underlying visuospatial skills might account for performance in the ROCFT in children and adolescents with ASD, compared with typically developing (TD), by considering the role of local/global visuospatial processing. The study involved 39 participants who have ASD without intellectual disability, and 57 TD aged 8–16 years. The participants were administered tasks assessing visuospatial organization abilities, manual dexterity, visual perception, mental rotation, spatial‐sequential, spatial‐simultaneous WM, and visuospatial processing. Our results suggest that manual dexterity and visuospatial processing similarly explain performance in both groups, while differences in visuospatial WM account for the two groups' visuospatial organization abilities. Spatial‐simultaneous WM predicted performance in copy and recall conditions in the TD group but not in the ASD group, while spatial‐sequential WM only did so in the latter group, reinforcing the tendency of children with ASD towards local bias in the visuospatial organization domain. The implications of these findings are discussed.

**Lay Summary:**

The visuospatial organization abilities of children and adolescents with and without autism were compared, considering their underlying visuospatial skills. Visuospatial organization impairments emerged for children with autism, who differed from typically developing children in the underlying visuospatial skills involved. Given the crucial role of visuospatial organization abilities in everyday life, our results could inspire practitioners to develop training interventions that take into account the strengths and weaknesses of individuals with autism.

## INTRODUCTION

The Rey–Osterrieth complex figure test (ROCFT; Rey, 1941, 1968) is a widely used neuropsychological test introduced by Rey as a measure of visual spatial construction and memory (Rey, 1941; Shin et al., 2006; Swenson et al., 2013). Studies in literature have shown that ROCFT involves different cognitive abilities including attention, visual perceptual abilities, visuomotor coordination, working memory, as well as executive functions (Batjes, 2019; Freeman et al., 2000; Schreiber et al., 1999; Shin et al., 2006; Somerville et al., 2000). Despite its complex nature, the salience of visuospatial and organizational skills in performing the ROCFT has been highlighted in previous studies, allowing the definition of this task as a visuospatial organization measure (Fujii et al., 2000; Smith & Zahka, 2006).

Many studies have identified certain peculiarities in the way complex visual stimuli are processed by individuals with autism spectrum disorder (ASD), especially those without intellectual disability (Caron et al., 2006; Kuschner et al., 2009). ASD is a lifelong neurodevelopmental condition characterized by deficits in social communication and interaction, alongside obsessive/stereotyped patterns of behavior, interests, or activities (APA, 2013). A preference for locally oriented processing has often been reported as a core feature of individuals with ASD, in line with the Weak Central Coherence theory (Happé & Frith, 2006) and the Enhanced Perceptual Functioning model (Mottron et al., 2006). Both theories attribute a local processing style to individuals with ASD, but focus on different underlying reasons: a global processing deficit according to the former theory; and an enhanced local processing ability according to the latter (McKenzie et al., 2018). No clear consensus has emerged, however, on the local or global processing styles of individuals with ASD (Van der Hallen et al., 2015). It has been suggested that we need to rethink the concept of local or global processing as a property of specific cognitive domains, rather than a central mechanism (D'Souza et al., 2016).

When the role of these processing strategies was examined in the visuospatial domain, a profile of strengths and weaknesses emerged for participants with ASD that depended on the visuospatial domain tested (e.g., Cardillo et al., 2020; Edgin & Pennington, 2005; Happé & Frith, 2006; Kuschner et al., 2007). In other words, children with ASD may operate both locally and globally, depending on the task and sub‐domain involved (Cardillo et al., 2018; D'Souza et al., 2016). Interesting findings for visuospatial organization tasks point to the importance of investigating global and local processing styles in this domain (Mammarella et al., 2019).

### Assessing visuospatial organization abilities using ROCFT in ASD

The ROCFT is widely used in research and clinical settings with both typically and atypically developing children (Salvadori et al., 2019). It enables visuospatial organization abilities and the use of local versus global processing strategies to be investigated. The task entails copying a complex geometrical figure and then reproducing it from memory few minutes later. Different scoring systems were developed to enhance their quantitative objectivity and/or in an effort to rate qualitative aspects of performance (see Mitrushina et al., 2005). The standard scoring system (Rey, 1968) is the most widely used for judging accuracy in the ROCFT: each of the 18 elements comprising the figure is rated from 0 to 2 on how accurately it has been drawn and on its positioning in the figure.

Qualitative aspects are often taken into account as well (see Troyer & Wishart, 1997) as they may help clinicians and researchers to identify local/global processing styles. When asked to reproduce the complex figure, some individuals begin by drawing its external elements (suggesting their use of a global strategy), while others start with internal elements (adopting a local approach) (Ropar & Mitchell, 2001). Booth's scoring procedure (2006) takes some qualitative aspects into account, assessing them using a quantitative approach. It considers the order of construction, in terms of the relative number of global as opposed to local features drawn in the initial stage of the drawing. Then six components of the ROCFT are rated for style, based on the degree of continuity in the drawing process. This scoring method enables researchers to identify a local bias in an individual's drawings. Another approach is taken in the Boston Qualitative Scoring System (BQSS; Stern et al., 1994), which is mainly used in research on adults' executive functioning.

Despite its validity in measuring visuospatial organization abilities and detecting processing strategies, the ROCFT has received little attention in research on the sphere of ASD, and findings have been mixed. Some studies identified impairments in the recall condition in ASD compared with typical development (Cardillo et al., 2018; Cardillo et al., 2020; Nydén et al., 2010; Prior & Hoffmann, 1990). Others reported no difference in the overall performance between individuals with ASD and their TD counterparts (Chan et al., 2009; Jolliffe & Baron‐Cohen, 1997; Ropar & Mitchell, 2001; Zandt et al., 2009). Kuschner et al. (2009) found no difference between children with ASD and TD control groups, as both used a locally oriented approach in reproducing the complex figure. Differences emerged when adults were tested, however, reflecting the acquisition of a more global processing with age in the typical life‐path. It is worth adding that many studies administering the ROCFT to investigate visuospatial performance used a modified version of the task, and failed to apply an objective scoring method to detect local/global processing (Chan et al., 2009; Zandt et al., 2009).

Given the diverse results, it could be useful to examine which visuospatial processes are engaged when performing the ROCFT, distinguishing between the copy and the recall of the complex figure. This could contribute to a better understanding of which abilities underpin local or global processing styles, and thus broaden our knowledge of how complex visual stimuli are processed by individuals with ASD compared with their TD counterparts.

### Visuospatial processes underlying the reproduction of the ROCFT

Visuospatial organization and drawing abilities are closely related to the maturation of other cognitive visuospatial skills, such as visuomotor coordination, perceptual abilities, mental rotation skills and working memory (WM) (Morra & Panesi, 2017; Panesi & Morra, 2016; Trojano et al., 2004; Van Gilder et al., 2021). This applies to an individual's performance in both copy and recall in the ROCFT, in which these abilities have revealed an important role (Fastame, 2020; McManus et al., 2010; Senese et al., 2015).

In a sample of TD children, Senese et al. (2015) highlighted the direct contribution of perceptual abilities, mental representation skills (i.e., mental rotation, complex figure identification), and WM when copying the complex figure, whereas its recall was only predicted by copying performance, which fully mediated the effect of visuospatial mental representation abilities. Trojano et al. (2004) likewise found that mental rotation significantly correlated with the copy condition in adults administered the ROCFT. Zappullo et al. (2020) later suggested instead that mental rotation does not influence ROCFT performance in TD children, unlike the case of the block design task.

The role of different visuospatial WM components in predicting ROCFT performance in TD children was underscored in a subsequent study showing that active visual WM predicted copying accuracy in the ROCFT, while copying performance and active spatial‐simultaneous WM abilities accounted for recall accuracy (Fastame, 2020). Trojano et al. (2004) found that some simple visual perception and visuospatial processing tasks, including line orientation judgments, significantly correlate with the copy condition when the ROCFT was administered to healthy adults.

Finally, several studies found visuospatial organization skills, as measured with the ROCFT, closely connected with visuomotor control and manual dexterity (Frisk et al., 2005; Van Gilder et al., 2021; Weber et al., 2013). The final drawing would therefore be the product of an interplay between various visuospatial competences.

To the best of our knowledge, no research has systematically focused on the visuospatial processes underlying ROCFT performance in children with ASD, comparing them with TD children.

### The present study

Based on previous findings, obtained mainly in TD children and adults, the cognitive processes that seem to be engaged in the ROCFT are: manual dexterity, perceptual abilities, mental rotation skills, visuospatial WM and visuospatial processing. Our study aimed to disentangle the relationships between these processes and the ROCFT in children with ASD but no intellectual disability (ID), by comparison with TD children. We therefore first looked for similarities and differences in ROCFT performance between the two groups of children. Then we considered which underlying visuospatial processes might account for the two groups' performance. The role of local/global visuospatial processing in the task's completion was also considered.

Using a slightly modified version of Booth's procedure (Lopez et al., 2008), previous research (Cardillo et al., 2018) had shown that useful information could be gleaned from the ROCFT about processing style in ASD. This procedure was consequently adopted in the present study to obtain in‐depth information about the processing style of our two groups. Though we expected some differences between them, inconsistencies in previous reports on ROCFT performance in children with ASD (see for example Cardillo et al., 2018; Cardillo et al., 2020; Cardillo et al., 2020; Nydén et al., 2010; Ropar & Mitchell, 2001; Zandt et al., 2009) prevented us from making any specific predictions regarding our groups' accuracy in ROCFT copy and recall. We expected different spatial abilities to explain the two groups' different performance in the ROCFT because children with ASD differ from TD children in specific visuospatial domains with a tendency towards local bias (Caron et al., 2006; Shah & Frith, 1993). As concerns their processing style, we expected a local bias with a fragmented drawing style to emerge for the ASD group (Cardillo et al., 2018; Nydén et al., 2010).

## METHODS

### Participants

The study involved 96 participants ranging in age from 8 to 16 years: 39 (34 M, 87%) with ASD but no ID, and 57 (45 M, 79%) TD controls. The two groups were matched for chronological age [*F*(1, 94) = 0.409, *p* = 0.524; Cohen's d = 0.13], genderdistribution [*χ* ^2^(*df =* 1) = 1.08, p = 0.299; Cramer‐V = 0.106], and full scale intelligent quotient (FSIQ) [*F*(1, 94) = 0.409, *p* = 0.524; Cohen's d = 0.13]. Only children who achieved a standard score of 80 or above for FSIQ on the Wechsler Intelligence Scale (WISC‐IV; Wechsler, 2012) were included in the sample. Table 1 shows a summary of the participants' characteristics.

**TABLE 1:** Characteristics of the two groups: Descriptive statistics and statistical analyses for individuals with autism spectrum disorder (ASD) and typically‐developing (TD) individuals

Measures	ASD (*n* = 39)	TD (*n =* 57)	*F* (1, 94)	*p*	Cohen's d
Age (months)					
Mean (*SD*)	139.64 (31.66)	143.98 (33.31)	0.409	0.524	0.13
Range	94–202	97–200			
IQ					
Mean (*SD*)	102.17 (15.21)	103.92 (11.58)	0.409	0.524	0.13
Range	80–135	83–132			
ADI‐R:A (reciprocal social interaction)					
Mean (*SD*)	15.49 (7.34)	3.70 (2.43)	127.3	<0.001	2.17
Range	0–28	0–8			
ADI‐R:B (language/communication)					
Mean (*SD*)	11.56 (4.90)	3.14 (2.15)	132.0	<0.001	2.22
Range	1–19	0–10			
ADI‐R:C (repetitive behaviors/interests)					
Mean (*SD*)	6.49 (3.14)	1.38 (1.54)	111.2	<0.001	2.07
Range	1–14	0			

*Note*: IQ, intelligence quotient on the Wechsler Intelligence Scale for Children–IV (Wechsler, 2003) or Wechsler Adult Intelligence Scale–IV (Wechsler, 2008); ADI‐R, Autism Diagnostic Interview–Revised (Rutter et al., 2005).

The control group consisted of typically‐developing children and adolescents of normal intelligence with no history of psychiatric, neurological or neurodevelopmental disorders. They were enrolled and tested individually at school. The clinical group was enrolled via local community contacts, at centers specializing in neurodevelopmental disorders. All participants in the clinical group had been previously diagnosed according to the DSM‐IV‐TR (APA, 2000) or ICD‐10 (WHO, 1992) criteria at centers specializing in ASD. Their diagnosis was also confirmed with the Autism Diagnostic Interview ‐ Revised (ADI‐R; Rutter et al., 2005). Only participants who scored above the cut‐off on the three modules of the ADI‐R, including stereotyped behaviors, were considered. Children with ASD who were taking medication, or who had other known genetic conditions, neurological diseases, or physical disabilities were excluded.

All participants were native Italian speakers, and none had any visual or hearing impairments. Ethical approval was obtained from the research ethics committee at the University of Padova. All participants assented to their participation in the study, and their parents gave their written informed consent.

### Materials

#### Visuospatial organization abilities

The Rey–Osterrieth complex figure test (ROCFT; Rey, 1941, 1968) is a classical neuropsychological test, mainly involving visuospatial organization skills. Participants were first asked to reproduce a complex geometrical figure by copying it freehand. Then, after an interval of 3 min, they were asked to reproduce it from memory. Participants were given a blank sheet of paper to draw the figure on, and several pencils of different colors (one at a time) so that the examiner could track the drawing sequence. The standard scoring system (Rey, 1968) was used to judge the accuracy of the drawings, scoring each of the 18 elements comprising the figure from 0 to 2 based on their presence, accurate reproduction, positioning and orientation. Raw scores were considered for each participant: the higher the score the better the performance.

The ROCFT was also scored according to Booth's (2006) slightly modified scoring procedure (described by Lopez et al., 2008), which takes into account the order and style of the drawings. To compute the order of construction score, the first third drawn elements of the ROCFT were categorized and then assigned different weightings from 0 to 4, depending on whether it was a local internal (0 points) or perimeter element (1 point), a global internal (3 points) or an external element (4 points). The order index score ranges from 0 to 3.3. The style index was defined in terms of the degree of continuity in the drawing of six elements. The style index score ranges from 0 to 2. The order and style indexes were then computed and added together to obtain the Central Coherence Index (CCI), which could range from 0 to 2. A high score on the CCI is indicative of a global and continuous processing of the figure, while a low score indicates a local and fragmented processing style. Cronbach's *α* = 0.80.

#### Manual dexterity

Fine motor abilities were assessed using the Manual Dexterity 3 (MD3) of the Movement ABC‐2 (Henderson et al., 2007). Manual dexterity is the fine motor control of hands and fingers needed to manipulate objects. In line with the instructions manual, participants were administered the version of the subtest appropriate for their age band. They were asked to draw a trail between two lines marking a path, which was wider for the 7‐ to 10‐year‐olds, and narrower for those aged 11–16 years. The best of two trials was used to rate the task, taking the number of errors into account. The raw scores were standardized for age and gender using normative data. Cronbach's *α* = 0.77.

#### Visual perception

The Beery Visual Perception task is a supplemental task of the developmental test of visual‐motor integration (VMI; Beery, 2004) and is a paper‐and‐pencil test used to assess visual perception skills. The child is asked to identify each item's identical match from a set of similar shapes. The test includes 27 geometric shapes of increasing complexity. The score is the number of successful matchings. The raw scores were standardized for age and gender using normative data. The test–retest reliability is 0.88.

#### Mental rotation

The animal rotation (AR) task, adapted from Kaltner and Jansen (2014), is a paper and pencil task used to assess mental rotation abilities. Participants were instructed to look at a target figure and choose the corresponding figure from among four rotated options presented alongside. The stimuli consisted of 21 two‐dimensional figures of animals. Participants had 5 min to complete the task, and only accuracy was measured. One point was awarded for each correct answer. Accuracy was calculated as a proportion, the higher the proportion the better the performance. Cronbach's *α* = 0.87.

#### Visuospatial working memory

Two computerized tasks, adapted from Mammarella et al. (2018), were used to measure spatial‐simultaneous and spatial‐sequential WM. A 5 × 5 grid appeared on the screen and participants were asked to memorize a number of cells that became gray simultaneously or sequentially. In both tasks, the number of cells shown in each grid ranged in span from 2 to 8, with three trials for span level. A maximum of 21 trials (a total of 105 stimuli) were administered for each task, using a self‐terminating procedure. The task ended when participants failed to correctly identify two up to three trials of that particular span level. After 3 s the stimulus disappeared, and participants were shown a blank grid in which they had to click to reproduce the previously‐seen pattern of cells. No time‐limit was set for the response. In the spatial‐simultaneous matrices (SSM), participants were only asked to recall the exact position of the stimuli, which were simultaneously presented, while in the spatial‐sequential matrices (SSQM), they had to recall the stimuli by considering both the exact location and the order of presentation. All tasks were of increasing difficulty. The score was calculated as a percentage, that is, the number of elements correctly located (and, for SSQM, also considering presentation order) out of the total number of items performed, multiplied it by 100, as this approach has proved more reliable and increases the predictive validity of WM tasks (see Conway et al., 2005; Giofrè & Mammarella, 2014). Cronbach's *α* = 0.89 for SSM, and 0.83 for SSQM.

#### Visuospatial processing

The arrows task is a subtest of the Nepsy‐II battery (Korkman et al., 2007), which assesses the ability to judge line orientation, to create and manipulate a mental representation of an object, and it involves co‐ordinate based mental imagery. For each of the 21 items, participants looked at an array of arrows placed around a target and chose the arrows that were pointing to the center of the target. One point was awarded for each arrow correctly identified. The total scores obtained by each participant were compared with the normative values and expressed as scaled scores. Cronbach's *α* = 0.76.

### Procedure

Participants were tested individually in a quiet room at specialized centers (ASD) or at school (TD) during two sessions lasting approximately 30 min each. The tasks were presented in a counterbalanced order. The experimenter provided instructions on each task, letting the participant practice with each task before starting the experiment. The computerized SSM and SSQM tasks were administered using a laptop computer with a 15‐inch LCD screen.

### Statistical approach

First, a series of univariate ANOVAs were performed to estimate differences between the groups in the ROCFT measures (copy and recall), MD3, VMI, AR, SSM, SSQM, and arrows (see Table 2). Effect sizes were also computed for all tasks, recording Cohen's d, which expresses the effect size of the pairwise comparisons between the groups for the factors considered.

**TABLE 2:** Descriptive statistics and statistical analyses by group for the measures used, in individuals with autism spectrum disorder (ASD) and typically developing (TD) individuals

		ASD		TD		*F* (1, 94)	*p*	Cohen's d
		*M*	*SD*	*M*	*SD*			
ROCFT	Copy	21.70	8.07	27.74	5.69	19.13	<0.001	0.86
CCI		1.01	0.26	1.13	0.28	4.74	<0.001	0.45
Style index		1.09	0.33	1.33	0.25	16.60	<0.001	0.85
Order index		1.54	0.51	1.49	0.60	0.13	0.71	0.07
ROCFT	Recall	12.09	8.41	16.56	6.87	8.16	<0.01	0.58
CCI		0.95	0.30	1.21	0.32	15.68	<0.001	0.82
Style index		0.88	0.40	1.23	0.43	16.15	<0.001	0.83
Order index		1.71	0.50	1.90	0.58	2.58	0.11	0.33
MD3		5.90	4.25	9.72	3.27	24.78	<0.001	1.00
VMI		94.28	19.15	100.56	17.28	2.80	0.09	0.34
AR		0.79	0.28	0.85	0.22	1.45	0.23	0.25
SSM		27.45	23.28	33.86	17.87	2.32	0.13	0.31
SSQM		24.31	16.08	29.17	14.98	2.29	0.13	0.31
Arrows		27.61	7.46	30.17	4.04	4.70	0.03	0.43

Abbreviations: AR, animal rotation; Arrows, arrows task; MD3, manual dexterity 3; ROCFT, Rey–Osterrieth complex figure test; SSM, spatial‐simultaneous matrices; SSQM, spatial‐sequential matrices; VMI, visual‐motor integration.

Second, in order to analyze the associations between the above mentioned measures controlling for the effect of participants' age, partial correlation analyses using Pearson's correlation coefficient were performed separately for each group.

Then two separate linear regression analyses were conducted to investigate the association between the dependent variables (first regression: ROCFT‐copy; second regression: ROCFT‐recall) and the hypothesized predictors (age, manual dexterity, visual perception, mental rotation, simultaneous visuospatial WM, sequential visuospatial WM, visuospatial processing). For both models, the main and interactive effect of Group (i.e., ASD, TD) was included as well. The adjusted R‐square was used to examine the fit of the models.

Correlation was used to concisely summarize the direction and strength of the relationships between our variables, while regression was applied to build two models to predict ROCFT performances from a set of visuospatial predictors. Regression goes beyond correlation by inferring relationships between variables, predicting the value of the dependent variable from a given value of independent variables (Shi & Conrad, 2009). In this sense, regression models tend to be much more explanatory than correlations. In particular, in dealing with predictors of ROCFT, there is the necessity to take into account the multiple underlying visuospatial processes in an aggregated way, because each of them could contribute to explain the variance of the whole model.

The data were analyzed using R (R Core Team, 2019). The “stats” package was used to run the regressions and the “effects” package was used to obtain the graphics (Fox, 2003).

## RESULTS

### Preliminary analyses

Table 2 shows the descriptive statistics and statistical comparisons between the two groups (ASD and TD). Regarding their visuospatial organization abilities, the two groups differed statistically on ROCFT Copy (*d* = 0.86), and ROCFT Recall (*d* = 0.58), the ASD group proving less accurate than the TD group. When the ROCFT was scored using a slightly modified version of Booth's (2006) method, the two groups differed statistically on the central coherence index (copy: *d* = 0.45; recall: *d* = 0.82). In particular, they differed on style (copy: *d* = 0.85; recall: *d* = 0.83), with the ASD group scoring lower than the TD children, while no statistically significant differences emerged for order of construction (copy: *d* = 0.07; recall: *d* = 0.33).

Considering the other measures of visuospatial abilities, the two groups differed statistically in the MD3 (*d* = 1.00) and arrows task (*d* = 0.43), the TD group performing better than the ASD group. No statistically significant differences emerged between the two groups for the remaining measures (i.e., VMI, AR, SSM, and SSQM).

### Correlation analyses

The results of the partial correlation analyses divided by group are given in Table 3. Pearson's correlation analyses have been performed to analyze the association between ROCFT (Copy and Recall), and the other visuospatial measures, controlling for the effects of participants' age. Results showed significant positive correlations between ROCFT copy and recall, and all the other measures for the ASD group. Differently, for the TD group, there were significant positive correlations between ROCFT copy, visual perceptual skills, spatial‐simultaneous WM visual processing skills (i.e., VMI, SSM, arrows), and between ROCFT recall, visual perceptual skills and spatial‐simultaneous WM (i.e., VMI, SSM).

**TABLE 3:** Partial correlational analyses (Pearson's correlation coefficients controlled for age), on visuospatial processing measures divided by group (autism spectrum disorder in the lower diagonal, and typical development in the upper diagonal)

	ROCFT copy	ROCFT recall	MD3	VMI	AR	SSM	SSQM	Arrows
ROCFT Copy	1	0.51**	0.12	0.38*	0.18	0.37*	0.11	0.27*
ROCFT Recall	0.66**	1	0.22	0.29*	0.06	0.41**	0.11	0.13
MD3	0.57**	0.52 **	1	0.21	0.12	0.24	0.47**	0.03
VMI	0.52**	0.37*	0.55**	1	0.30*	0.44**	0.44**	0.44**
AR	0.34*	0.34*	0.10	0.26	1	0.26	0.11	0.33*
SSM	0.34*	0.37*	0.44**	0.31	0.15	1	0.47**	0.19
SSQM	0.54**	0.46**	0.43**	0.39*	0.27	0.66**	1	0.18
Arrows	0.57*	0.47**	0.36*	0.44*	0.13	0.41*	0.44*	1

Abbreviations: AR, animal rotation; Arrows, arrows task; MD3, manual dexterity 3; ROCFT, Rey–Osterrieth complex figure test; SSM, spatial‐simultaneous matrices; SSQM, spatial‐sequential matrices; VMI, visual‐motor integration.

### Regression analyses

#### ROCFT copy

**FIGURE 1:**
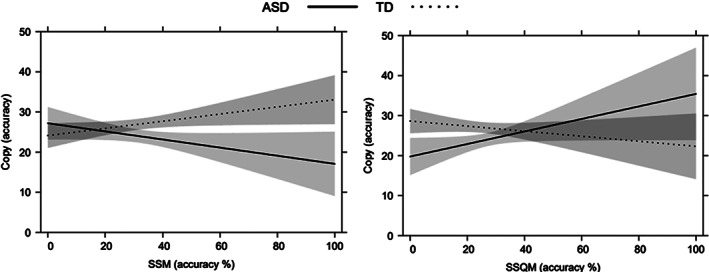
Significant interaction effects between group and visuospatial working memory (spatial‐simultaneous matrices [SSM] and spatial‐sequential matrices [SSQM]) on Rey–Osterrieth complex figure test (ROCFT) copy. Error bands represent 95% confidence intervals

Considering the linear regression model run for ROCFT Copy, our variables together accounted for 63% of the variance (*F*[14, 81] = 12.62, *p* < 0.001) calculated using the adjusted R‐squared (see Table 4). As concerns main effects, Age (*β* = 0.41, *t* = 4.01, *p* < 0.001), Group (*β* = 0.19, *t* = 2.61, *p* = 0.01), MD3 (*β* = 0.32, *t* = 2.58, *p* = 0.01) and Arrows (*β* = 0.25, *t* = 2.73, *p* = 0.008) were significantly associated with ROCFT Copy performance. Older participants and the TD group fared better, and higher MD3 and Arrows scores predicted a better ROCFT Copy performance. Two interaction effects ‐ the interactions between Group and the two visuospatial WM tasks ‐ were found statistically significant (SSM *β* = 0.36, *t* = 2.75, *p* = 0.007; SSQM *β* = −0.34, *t* = −2.28, *p* = 0.02). As shown in Figure 1, the two groups appeared to have a different visuospatial WM pattern when asked to copy the figure: a higher score in the SSM was consistent with a better in ROCFT Copy performance in the TD group, but not in the ASD group, where lower scores for the SSM predicted a better performance in the copy condition. On the other hand, higher scores for the SSQM coincided with better copying in the ASD group. No other statistically significant main or interactive effects emerged.

**TABLE 4:** Regression analyses with ROCFT copy and recall as dependent variables

ROCFT copy					
Predictors	*B*	Standardized beta	*SE*	*t*	*p*
Age (months)	3.07	0.41	0.76	4.01	**<0.001**
Group	2.83	0.19	1.08	2.61	**0.01**
MD3	2.43	0.32	0.94	2.58	**0.01**
AR	1.24	0.16	0.76	1.64	0.10
SSM	−2.11	−0.28	1.21	−1.74	0.08
SSQM	2.40	0.33	1.22	1.97	**0.05**
VMI	0.63	0.08	0.90	0.70	0.48
Arrows	1.93	0.25	0.70	2.73	**0.008**
Group*MD3	−1.92	−0.16	1.29	−1.49	0.14
Group*AR	−1.23	−0.11	1.08	−1.13	0.26
Group*SSM	3.98	0.36	1.45	2.75	**0.007**
Group*SSQM	−3.37	−0.34	1.48	−2.28	**0.02**
Group*VMI	0.56	0.05	1.15	0.49	0.63
Group*Arrows	−0.80	−0.06	1.33	−0.60	0.55

ROCFT recall					
Predictors	*B*	Standardized beta	*SE*	*t*	*p*
Age (months)	3.10	0.39	0.93	3.33	**0.001**
Group	0.88	0.05	1.32	0.67	0.50
MD3	2.67	0.34	1.15	2.32	**0.02**
AR	1.60	0.20	0.92	1.73	0.09
SSM	−0.52	−0.06	1.48	−0.35	0.73
SSQM	1.15	0.15	1.48	0.77	0.44
VMI	−0.41	−0.05	1.09	−0.37	0.71
Arrows	1.61	0.20	0.86	1.87	0.06
Group*MD3	−1.04	−0.08	1.57	−0.66	0.51
Group*AR	−2.42	−0.20	1.32	−1.84	0.07
Group*SSM	3.88	0.33	1.76	2.20	**0.03**
Group*SSQM	−2.87	−0.27	1.80	−1.59	0.11
Group*VMI	1.54	0.14	1.40	1.10	0.27
Group*Arrows	−0.87	−0.06	1.61	−0.54	0.59

*Note*: Statistically significant values (*p* <. 05) are shown in bold.

Abbreviations: AR, animal rotation; Arrows, arrows task; MD3, manual dexterity 3; ROCFT, Rey–Osterrieth complex figure test; SSM, spatial‐simultaneous matrices; SSQM, spatial‐sequential matrices; VMI, visual‐motor integration.

#### ROCFT recall

**FIGURE 2:**
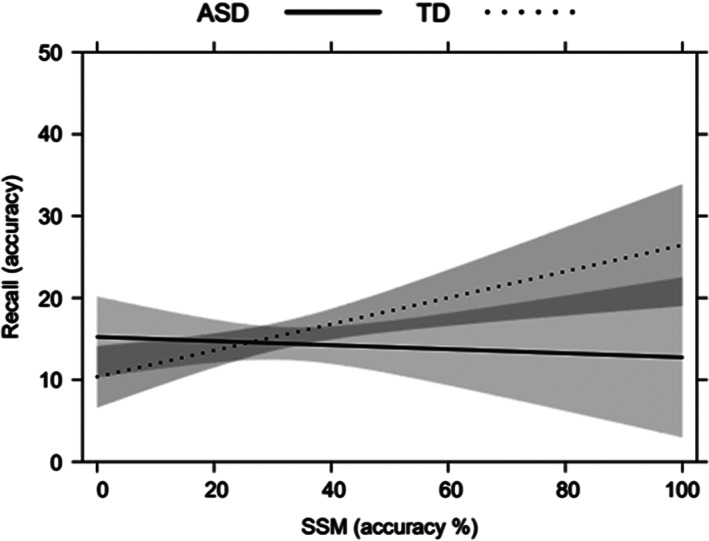
Significant interaction effects between group and spatial‐simultaneous matrices (SSM) on Rey–Osterrieth complex figure test (ROCFT) recall. Error bands represent 95% confidence intervals

In the regression model run for the recall condition, our variables accounted for 51% of the variance (*F*[14, 81] = 8.15, *p* < 0.001) calculated using the adjusted R‐squared (see Table 4). As concerns the main effects, Age (*β* = 0.39, *t* = 3.33, *p* = 0.001) and MD3 (*β* = 0.34, *t* = 2.32, *p* = 0.02) were associated with performance in ROCFT Recall. Older children and participants who fared better in the MD3 performed better in the recall condition. A significant effect of the interaction between Group and the SSM emerged as well (*β* = 0.33, *t* = 2.20, *p* = 0.03), showing that higher scores in the SSM predicted better performance in the recall condition for the TD group, but not for the ASD group (see Figure 2). No other statistically significant main or interactive effects emerged.

## DISCUSSION

The first aim of the present study was to analyze the visuospatial organization abilities of children and adolescents with ASD using the ROCFT, comparing their performance with that of a TD group. The role of local/global visuospatial processing in the task's completion was also considered. Participants' drawings were scored using two different procedures—the standard scoring system developed by Rey (1968), and Booth's method (described by Lopez et al., 2008)—to better explore the use of a local or global processing style.

Significant differences emerged between the two groups, the ASD group proving less accurate than the TD group in both ROCFT conditions (copy and recall). The findings of previous studies had been inconsistent: ROCFT performance was impaired in participants with ASD in some cases (Cardillo et al., 2018; Cardillo, Erbì, et al., 2020; Nydén et al., 2010; Prior & Hoffmann, 1990), while no significant differences for participants with ASD compared with TD had emerged in others (Chan et al., 2009; Jolliffe & Baron‐Cohen, 1997; Ropar & Mitchell, 2001; Zandt et al., 2009). Our findings therefore partially agree with previous research.

Local versus global visuospatial processing was considered here in an effort to shed more light on our participants' performance in this complex task, and to better explain the discrepancies vis‐à‐vis some earlier reports. Our results confirmed and extended previous findings (Cardillo et al., 2018) that the drawings of individuals with ASD featured a low coherence for both copy and recall. Order of construction and style indexes were examined to provide more information on our participants' drawings. No differences emerged between the two groups for order of construction, meaning that they did not differ in the number of local as opposed to global features drawn in the initial stage of the test. Consistently with previous reports, the children with ASD proved capable of operating both globally and locally (Cardillo et al., 2018; D'Souza et al., 2016). As for the style index, the ASD group scored lower than the TD group, revealing a more fragmented drawing style characterized by separately‐drawn components with clearly more disjointed lines, or elements drawn piecemeal. Taking these findings together with the central coherence index, participants with ASD would seem to opt for a locally‐oriented processing, in line with the weak central coherence theory (Happé & Frith, 2006) and the enhanced perceptual functioning model (Mottron et al., 2006). Our results are also consistent with previous reports of individuals with ASD showing a preference for a local processing style in the visuospatial organization domain (Cardillo et al., 2018; Nydén et al., 2010).

As regards the other visuospatial processing measures considered, the ASD group had more difficulty with visuomotor and visuospatial processing tasks. This is consistent with previous reports of impairments in manual dexterity (Lidstone et al., 2020; Liu & Breslin, 2013), and line orientation discrimination (Korkman et al., 2007; Narzisi et al., 2013) in individuals with ASD. Our two groups' performance was similar in terms of visual perception, mental rotation and visuospatial WM domains, however, as seen in previous studies (Cardillo, Erbì, et al., 2020; Green et al., 2016; Oliver, 2013; Ozonoff & Strayer, 2001; Rohde et al., 2018). It is worth noting that, as concerns visuospatial working memory performances, mixed results have been previously reported in ASD (Desaunay et al., 2020; Kercood et al., 2014; Wang et al., 2017). Furthermore, previous studies (e.g., Desaunay et al., 2020; Hamilton et al., 2018; Semino et al., 2021) suggested that executive functions and stimulus modality can contribute to group differences.

To further investigate performance in the ROCFT, we also taken into account the role of the underlying visuospatial processes. Our correlational analysis suggested positive significant correlations between ROCFT (both copy and recall) and all the other visuospatial measures in the ASD group, whereas for the TD group positive correlations emerged between ROCFT (both copy and recall) and visuospatial processing, visual perception and spatial‐simultaneous WM (Fastame, 2020; Senese et al., 2015; Trojano et al., 2004; Van Gilder et al., 2021). Additionally, we examined the association between the hypothesized visuospatial predictors (manual dexterity, perceptual abilities, mental rotation skills, visuospatial WM and visuospatial processing) and ROCFT performance, considering the copy and recall conditions separately, and also checked for a predictive role of age and group.

Taking the effect of all variables into account, we found a significant main effect of age, manual dexterity and visuospatial processing, and a significant interactive effect of group with the two visuospatial WM tasks in predicting ROCFT copy performance. Concerning the effect of age, our results confirmed that copying skills improve with time across childhood and adolescence, in both typical and atypical development (Gallagher & Burke, 2007). We also found that higher scores in manual dexterity and visuospatial processing tasks predicted greater accuracy in the copy condition. This is consistent with previous studies showing positive associations between copying performance and measures of visuomotor control and manual dexterity (Frisk et al., 2005; Weber et al., 2013) or simple visuospatial processing tasks (Trojano et al., 2004).

As in previous reports, we found a significant link between ROCFT copy and visuospatial WM (Fastame, 2020; Senese et al., 2015), but the two groups' copying performance was intriguingly predicted by a different visuospatial WM pattern. Higher scores in the spatial‐simultaneous WM task predicted a better performance in the TD group, whereas lower scores predicted a better performance in the ASD group; and higher scores in the spatial‐sequential WM task predicted a better copying performance, but only in the ASD group. As Fastame (2020) neatly explained, spatial‐simultaneous processes are related to pattern encoding (i.e., a type of processing that considers the spatial relationships of the elements comprising a pattern as part of a whole, integrated image). Spatial‐sequential processes are related instead to path encoding (i.e., a type of processing that allows for information to be represented sequentially, step by step) (see Lecerf & de Ribaupierre, 2005; Mammarella et al., 2008). Spatial‐simultaneous processes therefore seem to refer to the global processing of visuospatial stimuli, while spatial‐sequential processes have to do with their local processing. Bearing this in mind, our results indicate that different WM processing strategies sustained our groups' ROCFT copy performance. A global‐stimulus‐oriented approach (i.e., spatial‐simultaneous WM) proved useful for the TD group, while a local‐stimulus‐oriented approach (i.e., spatial‐sequential WM) was used by the group with ASD. In short, this latter clinical group took a less effective approach to the task. Here again, our findings are consistent with the hypothesis that individuals with ASD prefer a local processing style in the visuospatial organization domain (Cardillo et al., 2018; Nydén et al., 2010), whereas TD individuals benefit from the presentation of global rather than local stimuli, in line with the global dominance hypothesis (Navon, 1977). It is worth noting that our two groups did not differ in terms of visuospatial WM, and this further strengthens the hypothesis that a different processing style influenced their drawing performance. However, mixed results emerged in previous studies on the visuospatial WM abilities in ASD samples (Macizo et al., 2016; Ring et al., 2020). Our results suggest that it might be useful to consider local/global processing strategies to better explore the association between processing approaches and underlying spatial WM in ASD.

The results of our regression model for ROCFT recall partially overlap with those obtained for the copy condition, as there was a significant main effect of chronological age and manual dexterity, and a significant interactive effect of Group with spatial‐simultaneous WM. Higher scores for spatial‐simultaneous WM predicted a better ROCFT recall performance in the TD group, but not in the ASD group. This finding is consistent with the report from Fastame (2020), who also found that spatial‐simultaneous WM accounted for ROCFT recall accuracy. Here again, an advantage of using a global processing approach emerged for the TD group, consistently with the global dominance hypothesis (Navon, 1977).

To sum up, our participants with ASD performed less well than TD in the ROCFT, for both Copy and Recall. Manual dexterity and visuospatial processing similarly influenced both groups' performance, but different underlying visuospatial WM components might have a part to play as well. A different processing style seemed to underlie the two groups' drawing performance, with a global‐stimulus‐oriented approach proving more useful for the TD group, and a local‐stimulus‐oriented approach being used by the group with ASD.

It is worth noting that bivariate correlations and multivariate regression analyses showed different patterns of results in our ASD and TD groups. However, multivariate regression goes beyond correlation by inferring relationships between variables, predicting the value of the dependent variable from a given value of a set of predictors (Shi & Conrad, 2009). Thus, in this case, bivariate correlations must be taken cautiously, because they do not consider the multiple variability inside the model. Differently, regression models consider the multiple underlying processes in an aggregated way, which might be much more explanatory in light of the present study's aims.

Despite the novelty and significance of our findings, some limitations of the present study need be taken into consideration. One concerns the small number of female participants in our sample, which prevented any gender‐specific data analyses. To gather information on any gender‐related differences in ROCFT performance, future studies should try to collect data on larger numbers of females with ASD. Given the significant effect of age that emerged here, future studies could also benefit from a longitudinal design, to monitor any developmental changes in participants with typical and atypical development.

Our findings have also clinical and educational implications. They may encourage clinicians to consider the different roles of the processes needed to complete the ROCFT, and to investigate the different domains of visuospatial abilities that may underpin drawing performance. Elucidating the underlying predictors, and analyzing strengths and weaknesses in the ROCFT performance of children with ASD could help practitioners to set goals tailored to their specific needs, choosing the training activities best suited to reinforcing their visuo‐constructive skills (Mammarella et al., 2021). At educational level, a better understanding of the predictors of visuospatial organization and drawings abilities in children and adolescent with ASD can help educators devising activities tailored to the specific weaknesses of this clinical profile. The strong contribution of fine‐motor abilities, visuospatial processing and working memory, which emerged from our results, points to the potential value of educational activities not focused exclusively on visuospatial organization and drawing skills, but also involving these predictors, in order to reach better outcomes. Overall, children with ASD should be taught to go beyond details. Training underlying visuospatial abilities (e.g., visuospatial WM) could contribute to help children using local/global processing strategies more flexibly and more adaptively based on the tasks' requests.

In conclusion, our findings suggest that both similar (i.e., visuomotor and visuospatial processing skills) and different abilities (spatial‐simultaneous versus spatial‐sequential WM) might be involved in explaining ROCFT performance in children and adolescents with ASD and their TD peers. We also found evidence of these two groups adopting a different visuospatial processing style, with participants with ASD showing a preference for a locally‐oriented processing in their drawings. Children and adolescents with ASD may reveal strengths and weaknesses in performing the ROCFT which may differ from those of the typical population.

## CONFLICT OF INTEREST

The authors declare no conflicts of interest.

## Data Availability

The data that support the findings of this study are available from the corresponding author upon reasonable request.
